# Structural characterization of PPTI, a kunitz-type protein from the venom of *Pseudocerastes persicus*

**DOI:** 10.1371/journal.pone.0214657

**Published:** 2019-04-11

**Authors:** Seyede Elnaz Banijamali, Mehriar Amininasab, Davood Zaeifi

**Affiliations:** Department of Cell and Molecular Biology, School of Biology, College of Science, University of Tehran, Tehran, Iran; George Washington University, UNITED STATES

## Abstract

The main purpose of this report is to investigate the structural property and new potential function of PPTI (*Pseudocerastes Persicus* Trypsin Inhibitor), a kunitz-type protein with inhibitory effect against trypsin proteolytic activity. Besides kunitz-type serine protease inhibitors, PPTI shows clear-cut similarities with dendrotoxins (DTXs), the other kunitz-type protein subfamily. The most important reason is the presence of functionally important residues of DTXs at correspondingly the same positions in PPTI. As such, we proposed the new ability of PPTI for inhibiting voltage-gated potassium channels and consequently its dual functionality. At first, we determined the solution structure of PPTI via Nuclear Magnetic Resonance (NMR) spectroscopy. Then by homology modeling, we constructed the model structure of trypsin-PPTI complex to confirm the same interaction pattern as trypsin-BPTI at complex interface. Finally, by Brownian dynamics (BD) simulations of PPTI NMR derived ensemble structure as ligand against homology model of human Kv1.1 potassium channel as receptor, we evaluated the potential DTX-like activity of PPTI. The results of our study support the proposed dual functionality of PPTI.

## Introduction

Kunitz-type proteins are one of the most-studied protein families due to their diverse physiological functions and stable conformation. Members of this protein family are small globular proteins (about 60 amino acid residues in length) with native compact conformation and distinct arrangement of secondary structure motifs, known as kunitz domain [1]. Their overall conformation is stabilized via three intra-chain disulfide bonds with highly conserved pattern of C1-C6, C2-C4, and C3-C5 [2–4]. This compact and stable structure of kunitz domain could be the reason for its wide usage in proteins with extracellular destination [5], in both mono and multi-domain forms, during evolution [4,6].

Diverging evolution of kunitz-type proteins has been leading to the formation of homologous proteins with different physiological functions including kunitz-type serine protease inhibitors and dendrotoxins (DTXs) [6,7]. The overall tertiary structures of these homologues are almost the same, while their functional sites and amino acid compositions are dramatically changed [8–10]. As such, in kunitz-type serine protease inhibitors functionally important residues (residues of anti-protease loop) are located in completely different part of the molecules, in comparison with DTXs [11]. Interestingly, there are only a few reported proteins with both functions of inhibiting serine proteases and blocking potassium channels. The common characteristic of these proteins is to show one of their physiological functions weaker than the other one, and more importantly, almost all of them are effective potassium channel blockers which could weakly interfere with proteolytic activity of serine proteases [7,12].

The kunitz-type serine protease inhibitors could reversibly inhibit serine proteases via a slow-tight interaction pattern [13]. In Bovine Pancreatic Trypsin Inhibitor (BPTI), the well-studied member of kunitz-type trypsin inhibitors, the interacting residues are located within two exposed loops consisting residues T11 to I19 (the anti-protease loop) and G36 to R39. In trypsin-BPTI complex the critical P1 residue of BPTI, K15, located at the anti-protease loop, interacts with D191 and S197 in S1 pocket of trypsin [3].

On the other hand, DTXs act as a facilitator of neurotransmitter release from pre-synaptic neurons by blocking ionic current in voltage-gated potassium channels (Kv) [1,9,14–16]. For an effective interaction with Kv channels, a basic residue of DTXs interacts with both negatively charged (E353 and D431 in Kv1.1) and aromatic residues (Y379 in Kv1.1) of the pore region in Kv channels [11,17,18]. According to site-directed mutagenesis studies, this basic residue along with a hydrophobic residue are mostly responsible in making interactions with Kv channels, and simultaneous mutations of these two residues cause a great reduction (about 1000 times) in DTXs function against Kv channels [1,12]. In addition, the configuration of functional sites in DTX variants is in a way that the critical basic residue protrudes from the functional surface [11] and keeps a certain distance (about 6.6±1 Å) from the hydrophobic residue [17,18].

In our previous work [19], we introduced PPTI (*Pseudocerastes Persicus* Trypsin Inhibitor) as a new member of kunitz-type serine protease inhibitors. According to the sequence comparison outcomes, the functionally important residues of DTXs are correspondingly present in PPTI, which brings up the hypothesis of PPTI bi-functionality. To evaluate this hypothesis, we used in silico approach and performed a series of Brownian dynamics (BD) simulations between PPTI NMR structures and Kv1.1 modeled structure. The results are promising and demonstrate the possible blocking activity of PPTI against potassium channels, with the same mechanism as DTXs. In what follows, we report the results of structure determination of PPTI by solution NMR spectroscopy and protein docking between PPTI NMR structures and both trypsin and Kv1.1 potassium channel by homology modeling and BD simulation, respectively.

## Results

### NMR resonance assignment

According to the standard methods, manual amino acid spin systems identification and sequential assignment were performed based on homonuclear TOCSY and NOESY spectra. The sequential spin system connections were identified by observing H^α^_i_-HN_i+1_, HN_i_-HN_i+1_ and H^β^_i_-HN_i+1_ crosspeak NOEs in 150 ms NOESY spectrum in which the fingerprint region is shown in S1 Fig. Two histidine residues, H19 and H55, were identified via the cross peaks of their ring protons. Moving in both downstream and upstream directions and using the characteristics spin systems of alanine, aromatic and amide residues, we completed the sequential assignment, except for P68, the last residue at flexible C-terminal tail. The observed long range NOEs between H^α^-HN and H^α^-H^α^ of residues confirm the existence of hydrogen bond pattern of a two-stranded antiparallel β-sheet, S2 Fig. To solve the ambiguities associated with the NOE assignment of NOESY spectrum or spin system identification due to the shielding or desheilding effect of aromatic rings, we used the constructed homology model of PPTI as the guiding structure. The results of the spin systems identification were confirmed by the ^1^H-^13^C HSQC spectrum. The chemical shifts of the resonance assignment of PPTI at 293 K are presented in S1 Table and have been deposited in BioMagResBank (BMRB) database, BMRB ID 36197.

### NMR structure calculations

**Fig 1:**
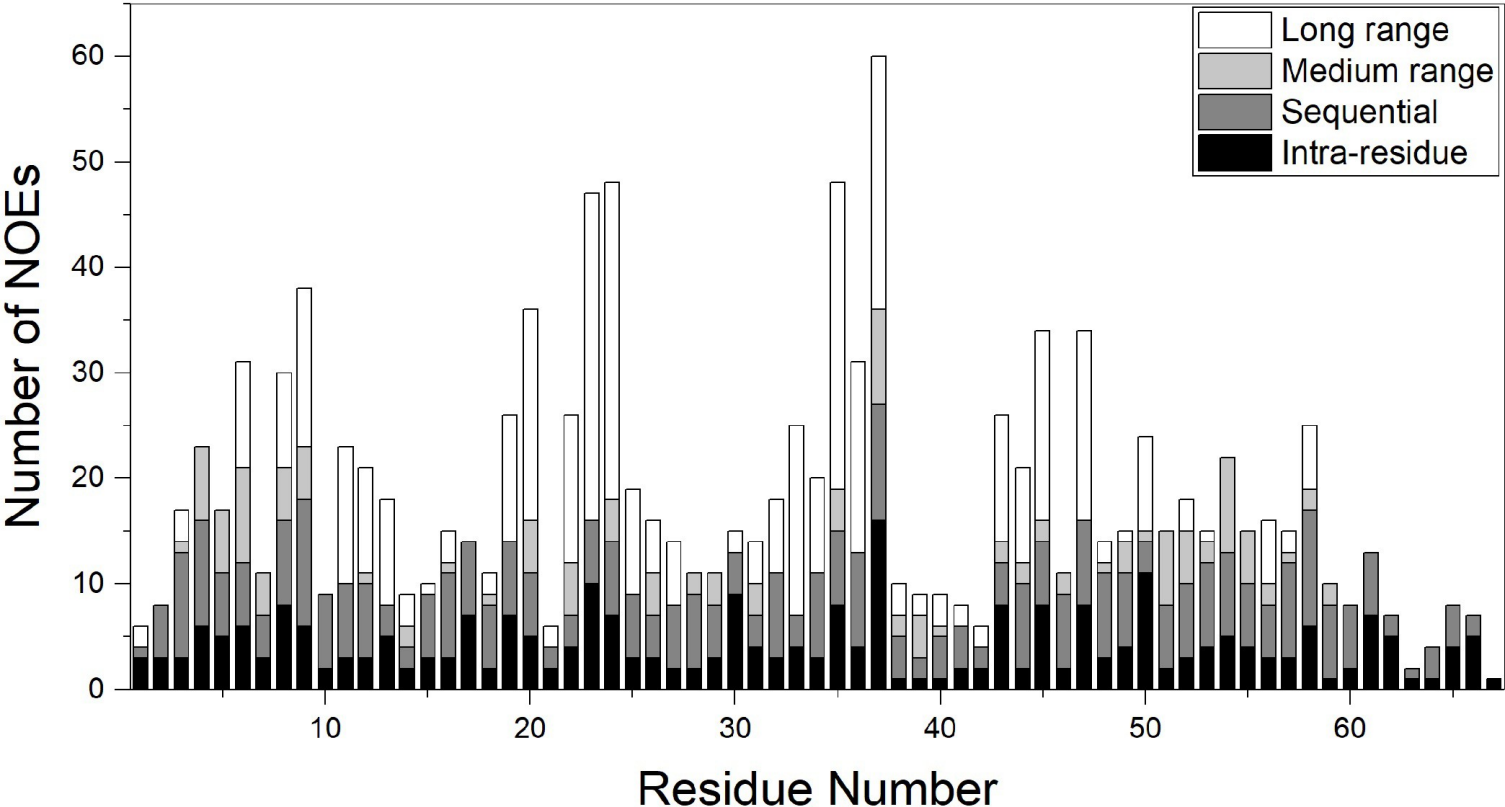
The number of NOEs per residue. In this chart the intra-residue, sequential, medium range and long range NOEs are determined in different compartments and respectively shown in black, dark gray, light grey, and white.

**Fig 2:**
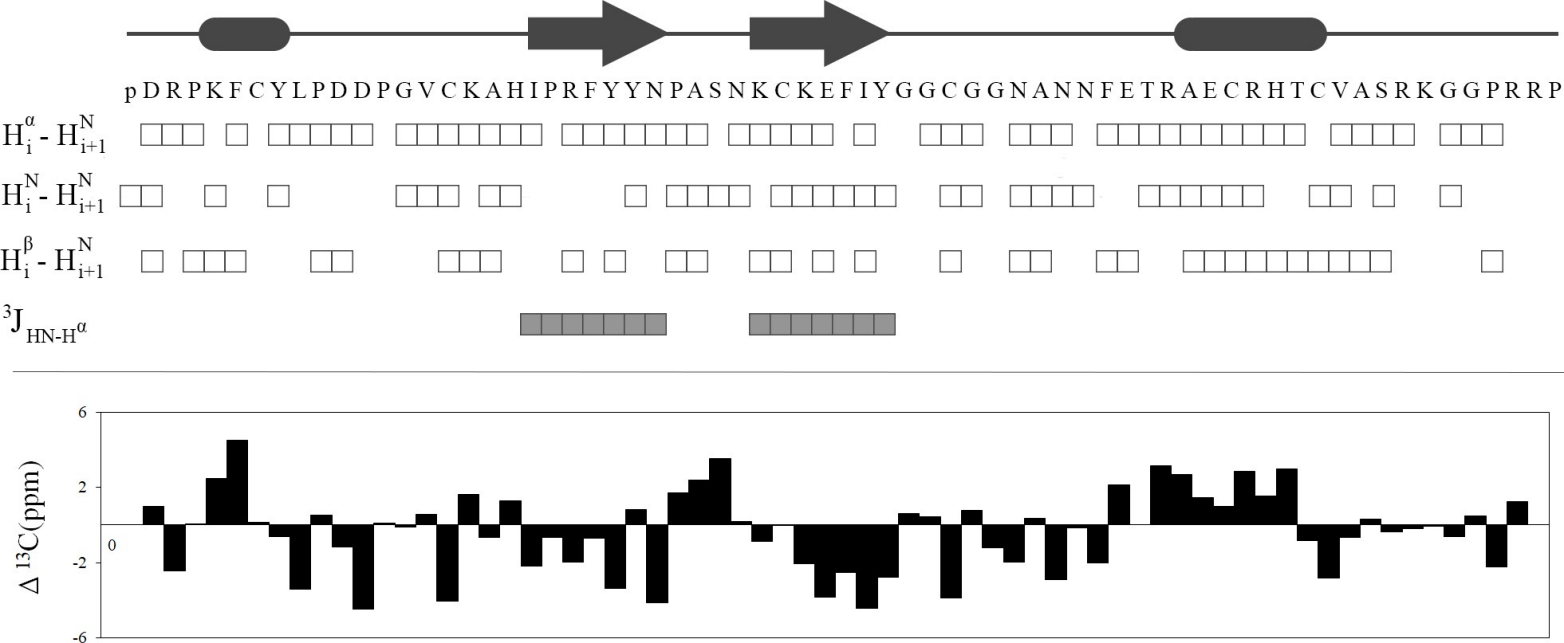
NMR data summary. At the top, the schematic view of predicted secondary structure motifs of PPTI, and the sequence of its amino acid residues are shown. The observed sequential NOEs and the ^3^J_HN-H_^α^ coupling constants that are used in structure calculation are respectively shown with open and filled squares. For sequential NOEs, in case of proline residues, HN refers to H^δ^ that was used for the sequential assignments. At the bottom, the chart of chemical shift deviations of C_α_ that were calculated with respect to random chemical shift values is presented.

Structure calculations were mainly based on the NOE distance restraints in which its sequence distribution is shown in Fig 1. In addition to NOEs, hydrogen bond distance restraints based on the preliminary structure calculations and observed NOEs (S2 Fig) along with dihedral angle restraints (S2 Table) were included in the final structure calculation. For the latter, we used the estimated ^3^*J*_HN-Hα_ of residues in β-sheet motif (with values > 8) and converted them into φ dihedral angle restraints. Moreover, by calculating the secondary chemical shifts of ^13^C^α^ atoms with respect to the corresponding random chemical shifts, and by using TALOS program suggestions, we adopted and imposed the restricted conformational search space for residues of secondary structure motifs. In Fig 2, the summary of NMR parameters for PPTI are presented.

**Fig 3:**
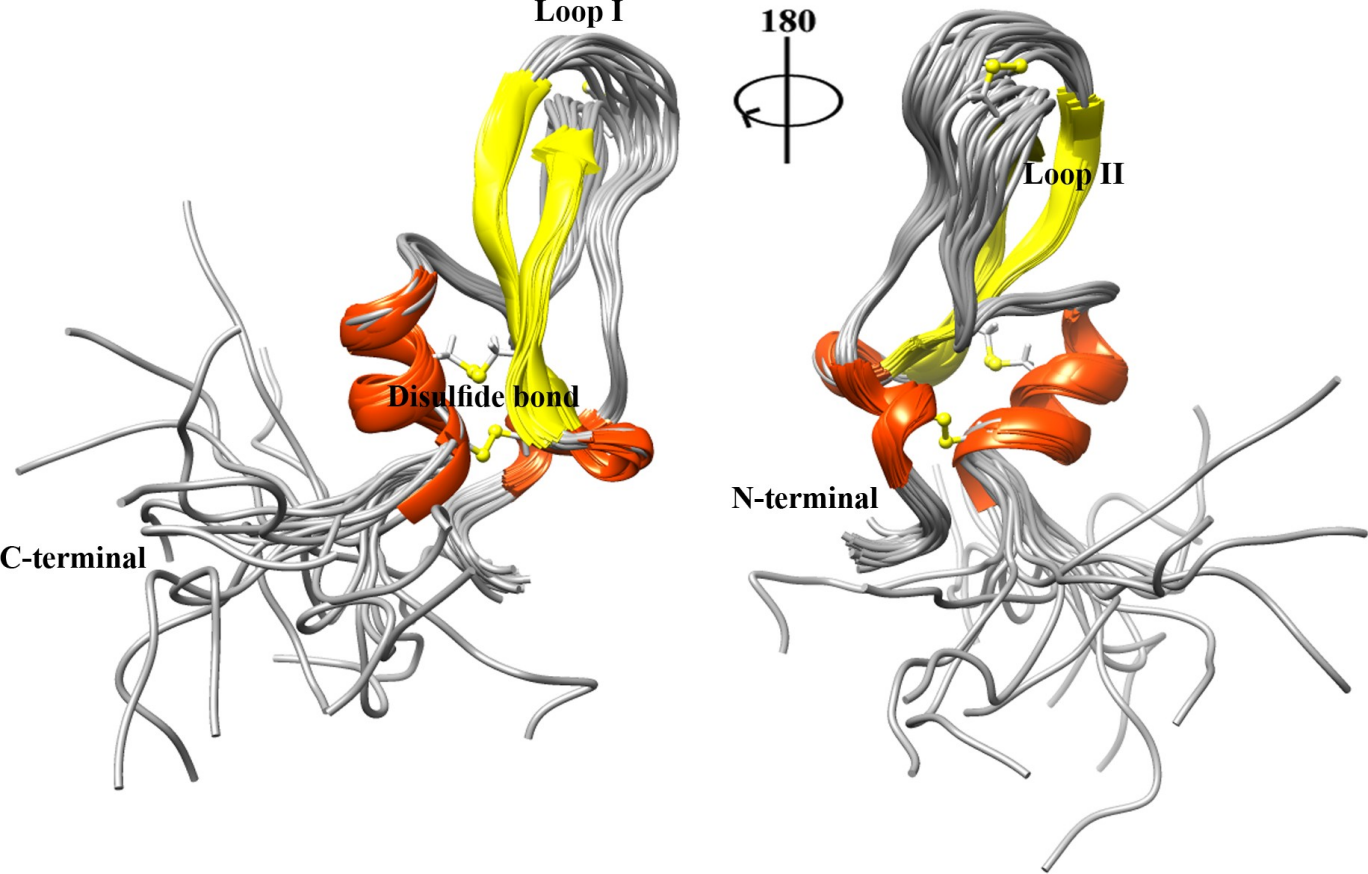
The ensemble of top twenty superimposed PPTI NMR structures. The structures are fitted to the best energetic one over the backbone atoms and the three intra-chain disulfide bonds are represented in sticks.

From 900 calculated structures in nine iterations of the standard protocols of ARIA and on the basis of energetic parameter, the best twenty refined structures of the last iteration were selected for further analysis. These final set of structures were geometrically evaluated and showed a good agreement with experimental restraints, with no NOE distance violations greater than 0.25 Å. The backbone dihedral angles were analyzed by PROCHECK-NMR and the resulted Ramachandran plots indicate that for all twenty best structures, 87% of nonglycine and nonproline residues are located at favored regions, and only 2.7% of residues are in disallowed regions (S2 Table). The twenty best energetic structures of PPTI are superimposed and displayed in Fig 3 and their coordinates were deposited in RCSB Protein Data Bank, PDB ID 6A5I.

### Structure description

PPTI, like most of the kunitz-type proteins, has a compact globular pear-shaped structure. According to the solved NMR structures, the secondary structure motifs of this protein and its arrangement are the same as the kunitz domain. There are two connecting loops in PPTI, named loop1 and loop2. Loop1 connects the N-terminal 3_10_ helix (residues K5 to Y8) to the first β-strand (residues I20 to N26) and contains functionally important residues of kunitz-type serine protease inhibitors (K17 and A18 as P1 and P1´ sites, respectively). Loop2, spanning from G38 to T49, connects the second β-strand (residues K31 to Y37) to the α-helix motif (residues A51 to C57) near the C-terminal end.

Three disulfide bonds in PPTI structure, on the other hand, connect both terminal ends (C7-C57); stabilize the orientation of two solution exposed loops with respect to each other (C16-C40); and localize and immobilize the C-terminal helical motif over the β-sheet (C32-C53).

According to the information from TOCSY and NOESY spectra, PPTI has a flexible disordered tail at its C-terminal part, consists of residues S60 to P68.

### Solvent accessibility of PPTI residues

**Fig 4:**
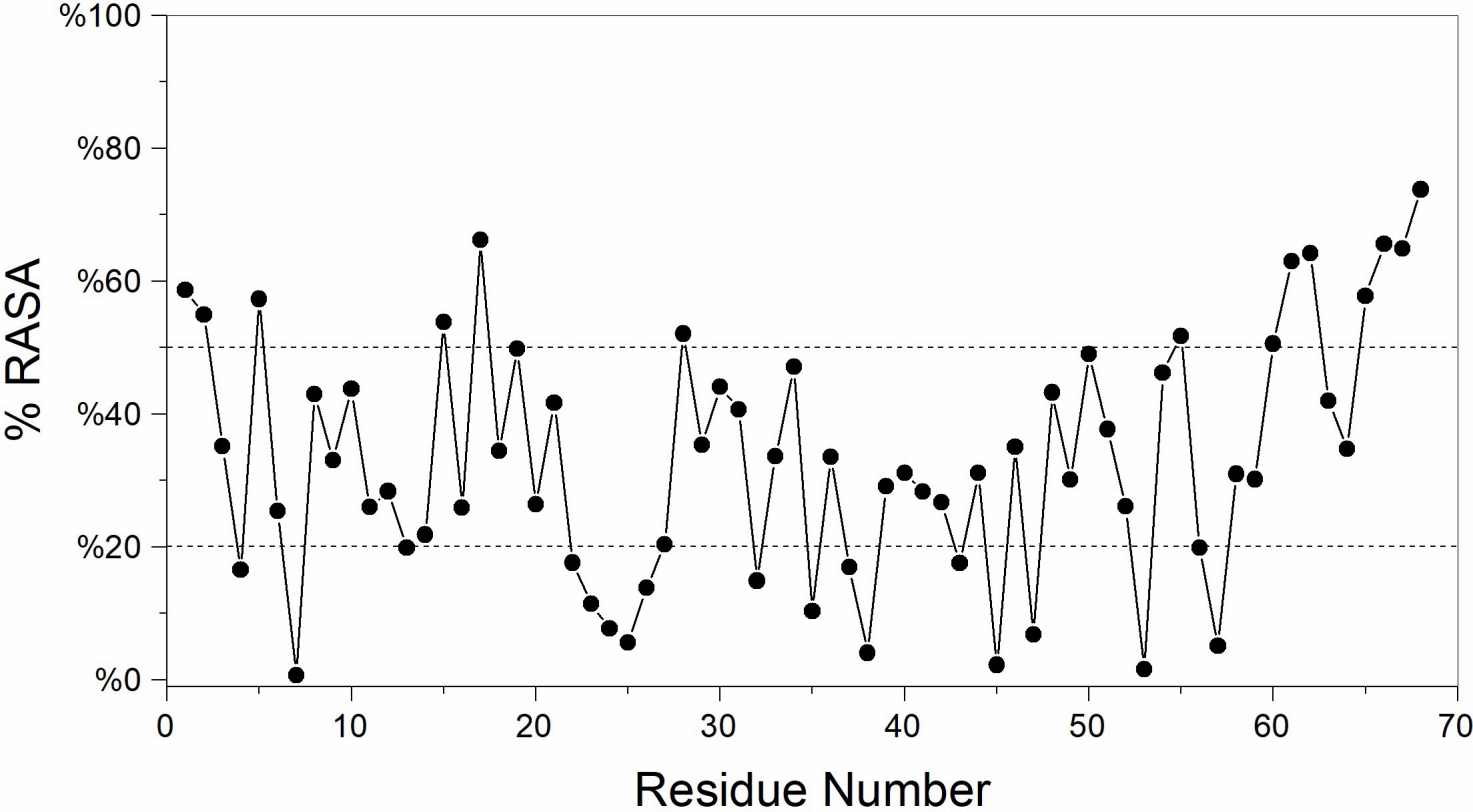
The relative solvent accessibility of PPTI residues. For each residue, the averaged value of relative accessible surface area (RASA) are calculated for the best twenty NMR structures. Both upper and lower limits that used for defining buried and solvent exposed residues are shown with dashed lines.

The accessibility of residues in the twenty best NMR structures of PPTI was evaluated by determining Relative Accessible Surface Area (RASA) for each residue (Fig 4). For calculating this value, at first the total accessible surface area of each residue in PPTI’s NMR structures were calculated, regardless of the neighboring residues, and averaged over the twenty top NMR structures. Next, the accessible surface area of residues for the twenty top NMR structures were calculated by regarding all residues. Finally, the ratio of the averaged accessible surface area of each residue to the averaged total accessible surface area of the corresponding residue defined as RASA. The resulted values are presented in percent scale in Fig 4.

By considering the value of 20% RASA as an upper limit for buried residues, 18 buried residues in PPTI have been found that approximately half of them are hydrophilic residues participating in the β-sheet motif. For assigning solution exposed residues, we set the lower limit of 50% for RASA. As such, residues with RASA values more than 50% are classified as solution exposed ones. Without considering the residues at both terminal ends, the most solution exposed residues are K5 and K17, which are critical in the attributed physiological functions of PPTI.

### Molecular modeling of the complex between PPTI and trypsin

**Fig 5:**
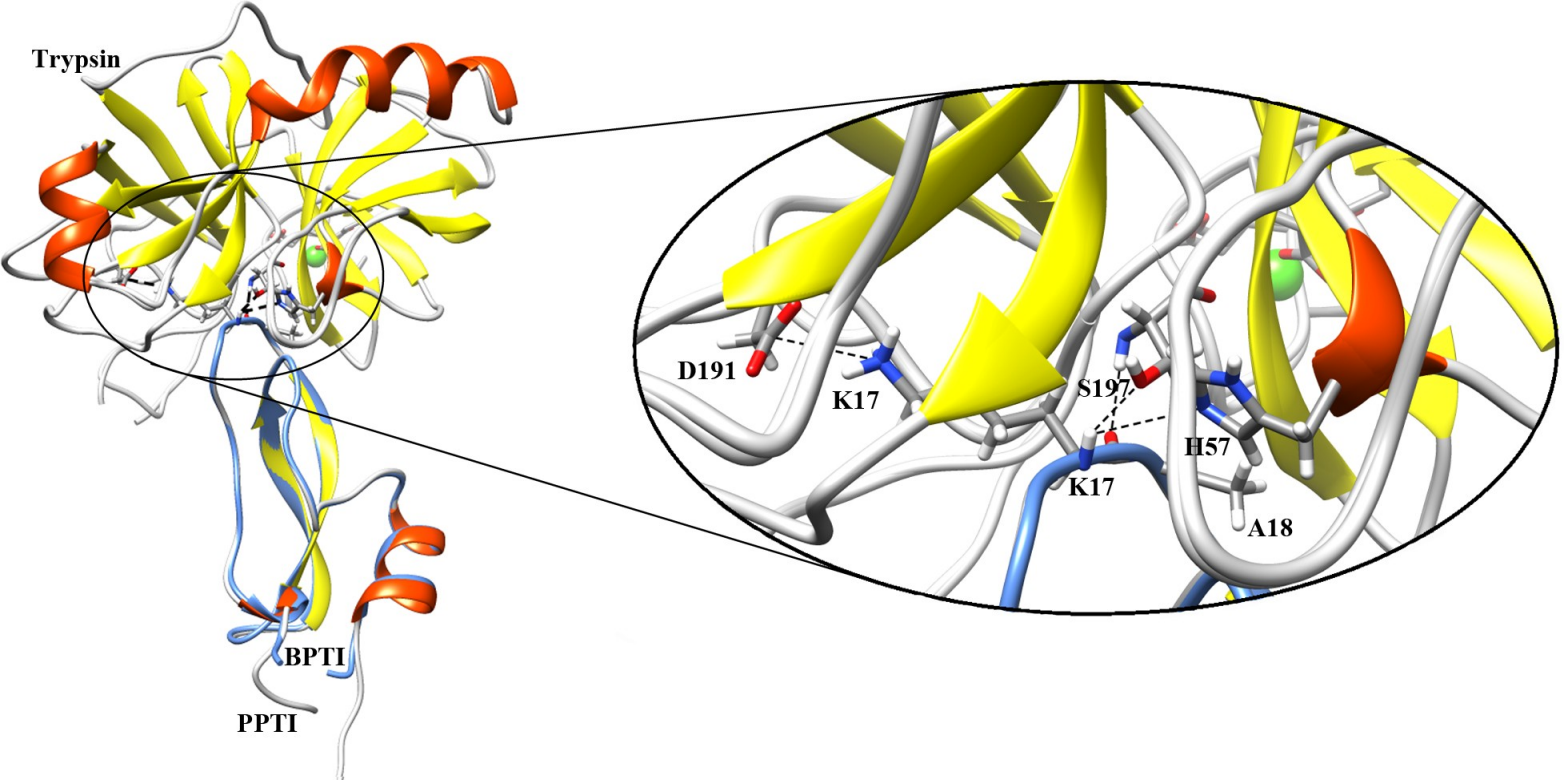
The modeled structure of trypsin-PPTI complex fitted to its template (trypsin-BPTI complex). On the right side, the close view of trypsin active site (S1 pocket) and anti-protease loop of both BPTI and PPTI are shown. The possible hydrogen bond and electrostatic interactions between labeled residues are shown by dashed lines.

The high resolution crystal structure of BPTI in complex with trypsin (PDB code 4Y0Y) was selected as template to model the corresponding complex with PPTI. By considering the DOPE score, the best model was analyzed for RMSD and binding residues at interface. The RMSD between the backbone atoms of BPTI and PPTI in complex with trypsin was about 0.2 Å representing a good compatibility between the template structure and the target model. As Fig 5 shows, residues V13 to P19 of PPTI form the binding interface with trypsin in the modeled complex. Similar to BPTI, PPTI has the characteristic lysine at P1 site (K17) that interacts with residues of trypsin in S1 pocket. Some of these important interactions are: i) a hydrogen bond between K17 backbone carbonyl oxygen to the main chain NH group of S197; ii) two hydrogen bonds between K17 NH group with both OG of S197 and NE2 of H57; iii) a simultaneous hydrogen bond and electrostatic salt bridge between the ε-amine group of K17 and the side chain carboxyl groups of D191.

### Molecular modeling of the 3D structure of human Kv1.1 potassium channel

Voltage-gated potassium channels are composed of four transmembrane subunits (α-subunit) that form the channel part; and four cytoplasmic subunits (β-subunit) that regulate the channel function [20–22]. Each α-subunit passes 6 times through the cytoplasmic membrane, which are correspondingly identified as S1 to S6. The first four transmembrane segments (S1 to S4) comprise the voltage sensing domain and the other two segments (S5 and S6) participate in formation of the pore region of channel [17,22,23].

In order to investigate the sequence guided possible interaction between PPTI and potassium channels, we constructed a homology model for the α-subunits (by ignoring the voltage sensing domain) of human Kv1.1 potassium channel in open conformation. For this, we used four template structures in which two crystal structures were from eukaryotic sources (Kv1.2 channel with PDB codes 2R9R and 3LUT) and the other two crystal structures were from bacterial organism (KCSA channel with PDB codes 1BL8 and 2A9H). The best modeled structure was selected based on Dope Score with its structure shown in S3 Fig.

### Brownian dynamics simulation of the interaction between PPTI and human Kv1.1

**Fig 6:**
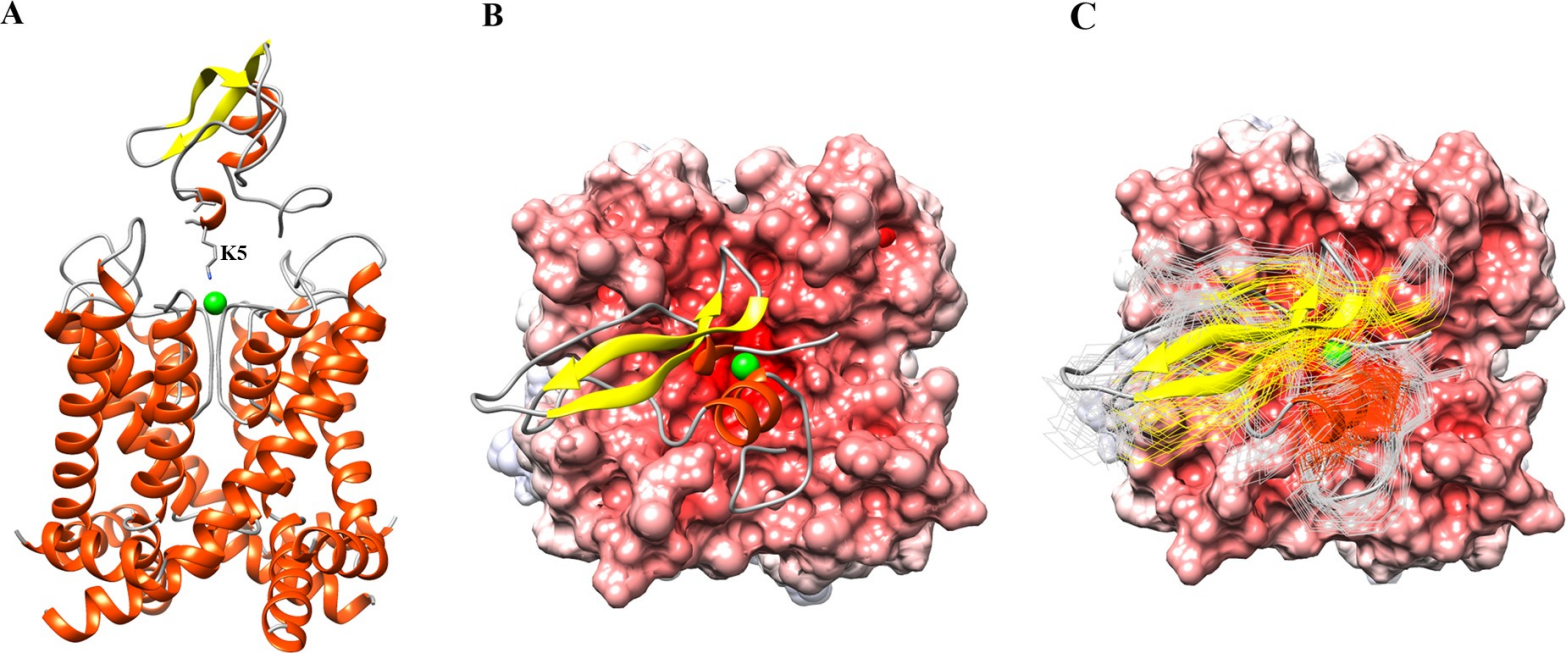
The modeled complex of PPTI and human Kv1.1 potassium channel. (A) The side view of the complex. The critical residue of PPTI (K5) and its orientation towards the pore region is labeled. The water molecule at the pore entry is shown as a green sphere. (B) The top view of the complex. The negative electrostatic surface potential of Kv1.1 is shown in red. (C) The top view of the best energetically modeled complexes between PPTI and human Kv1.1 potassium channel.

The mechanism of interaction between dendrotoxin variants and potassium channel is proposed as a physical blockage of the channel pore by DTXs. As such, the side chain of a critical lysine in DTX makes electrostatic and π-cationic interactions with negatively charged and hydrophobic residues in the pore region of potassium channel respectively [2,11,14,17,24]. In Kv channel variants, the critical residues that interact with inhibitors (like DTXs) are mostly located in the loop connecting S5 and S6, named as P-loop in literature. There is a highly conserved motif sequence in P-loop (TXGYGD, known as the signature sequence of Kv channels) which seems to be mainly responsible for making interaction with DTX homologues [14,22,23]. Thus for BD docking simulation, we selected nitrogen atom of ε-amine group of K5 residue from PPTI and oxygen atom of a water molecule which was placed at the pore entry of Kv1.1 with equal distances from four aspartic acid residues of the channel pore region, as the monitor atoms for detecting the formation of association. At the beginning in order to do a blind docking, we used the large distance criteria (15 to 30 Å) to find the better understanding of all possible docking orientations of PPTI relative to Kv1.1 in BD simulations. Interestingly and as we expected for the large monitor distances of association, the spatial distribution of center of mass of PPTI was mainly populated over the pore region of channel, S4 Fig. Then to have a better resolution, we set the monitor distance of association in decreasing manner from 10 to 5 Å in different simulations. In order to improve the rigid docking performance, a hundred uniformly sampled snapshot structures of PPTI from NPT MD trajectory (with 2.5 ns interval time) along with proposed structure for human Kv1.1 potassium channel were used as the initial structures for BD simulations. The interaction mode of the best energetic complex between PPTI and Kv1.1 potassium channel is shown in Fig 6A (side view) and Fig 6B (top view). As Fig 6A shows, the ε-amine group of K5 is in close contact with the water molecule at the pore region. In order to analyze the diversity of interaction modes, the fifty best energetic complexes are superimposed on each other (top view is shown in Fig 6C) and the RMSD of each PPTI structure was calculated with respect to the best energetic one and as Fig 6C shows, the overall interaction orientation of these fifty complexes are the same. The average value of RMSD was about 5.0 Å with minimum and maximum values of 1.3 and 8.4 Å, respectively.

**Fig 7:**
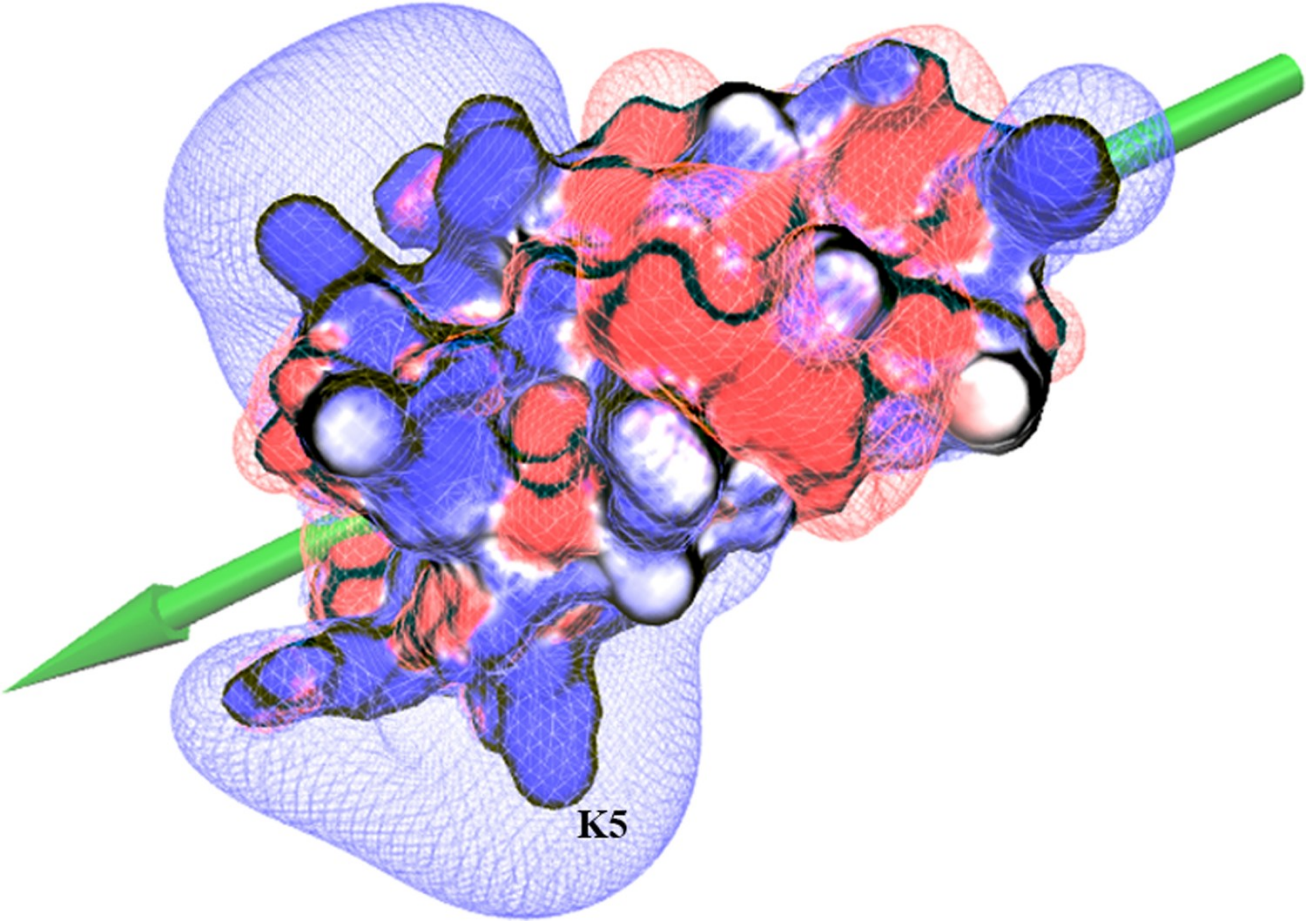
The electrostatic isocontour potential map and the surface potential of PPTI. The blue and red colors represent positive and negative fields respectively. The electric dipole moment vector of the protein is represented as a green arrow and the proposed critical basic residue of dendrotoxin activity (K5) is labeled.

For making a comparison, we performed similar BD simulations for α-DTX by considering its critical basic residue (i.e. K5), for which the best energetic complex is shown in S5 Fig. The average values of electrostatic interaction energy of five top complexes were about -11 and -7.5 kcal/mol for α-DTX and PPTI respectively. By assigning the total charge of +5 for PPTI and +8 for α-DTX at neutral pH and in order to account for the better electrostatic interaction energy of α-DTX toward Kv1.1 potassium channel, we calculated the associated electric dipole moment and electric field around these protein molecules (Figs 7 and S6, respectively) by VMD [25]. As these figures show, α-DTX has broader positive electric field and its dipole moment is better aligned with the direction of its critical basic K5 residue in comparison with PPTI.

## Discussion

In this research, we used both experimental and computational approaches for determining the structural and functional aspects of PPTI, a kunitz-type trypsin inhibitor extracted from venom mixture of the Persian false-horned viper. The best solved structures derived from solution NMR spectroscopy confirmed the presence of the motif structure known as kunitz domain in PPTI. Like kunitz-type proteins, the domain structure of PPTI is stabilized with the same topology of three highly-conserved intra-chain disulfide bonds. As a result, the both helical parts are brought in close contact with each other via the C7-C57 disulfide bond, and the α-helix in C-terminal part is located over the β-sheet motif via the C32-C50 disulfide bond. Also, PPTI has a flexible C-terminal tail, residues S60 to P68, which is not a common structural feature among small kunitz-type serine protease inhibitors and DTX variants.

Based on the calculated RASA indexes for the amino acids of PPTI, K5 and K17 are among the highly solution exposed residues that are proposed to be responsible for blocking potassium channels and inhibiting trypsin, respectively. The anti-trypsin activity of PPTI was experimentally determined [19] and the constructed model structure of trypsin-PPTI complex revealed the details of this interaction. Like BPTI, the P1 residue of PPTI (K17) contributes critically in interactions with the key residues of trypsin S1 pocket (i.e. H57, D191, and S197). Next, for evaluating the ability of PPTI in blocking potassium channels we performed a series of BD simulations between PPTI and human Kv1.1 potassium channel, and compared the mechanism of interaction with α-DTX, a well-known dendrotoxin. The results of this part show that PPTI could block Kv1.1 potassium channels with the same mechanism as dendrotoxins. Therefore, we can speculate the possibility of dendrotoxin-like activity of PPTI against Kv1.1 potassium channels, which makes PPTI as a possible bi-functional protein. Interestingly, almost all of the reported bi-functional kunitz-type proteins between serine protease inhibitors and dendrotoxins are effective potassium channel blockers with weak anti-protease activities in which PPTI is different [7,12]. Although the results of our in silico evaluation are promising, further experimental evaluations about PPTI’s dendrotoxin-like activity are needed in order to prove its dual-functionality.

## Materials and methods

### Molecular modeling of the 3D structure of PPTI

In order to overcome the ambiguity in identifying NMR derived restraints, the 3D structure of PPTI was modeled by comparative homology modeling according to our previous work [19].

### Sample preparation for NMR spectroscopy

PPTI was purified from the venom of Persian false-horned viper, *Pseudocerastes* persicus, according to the procedure described previously [19] and lyophilized by freeze dryer. The lyophilized PPTI protein was dissolved in H_2_O/D_2_O (90:10 v/v) pH 4 or 99.9% (v/v) D2O to reach the final concentration of 2 mM.

### NMR spectroscopy

All NMR experiments were performed on a Bruker DRX-500 spectrometer equipped with z-gradient ^1^H-^13^C-^15^N triple-resonance TXI probe. NMR spectra were acquired at 293 K. The two-dimensional homonuclear TOCSY [26,27] (75 ms mixing time), NOESY [27] (80 and 150 ms mixing times) and DQF-COSY [28] spectra and a natural abundance heteronuclear ^1^H-^13^C HSQC [27] spectrum were recorded with 2048 data points in direct t_1_ dimension and 512 increments in indirect t_2_ dimension. One zero filling was performed to obtain the final 1024×4096 data sets. Water suppression was achieved by applying WATERGATE pulse sequence or presaturation. The carrier frequency was set with respect to the center of residual water signal and DSS (2,2-dimethyl-2-silapantane-5-sulfunate) was used as an internal reference. All spectra were processed by XWIN-NMR software and analyzed with Sparky [29].

### NMR derived restrains

Spin system identification and sequential assignment were carried out according to the standard methods [30]. The interproton cross-peak intensities were measured from the 150 ms mixing time NOESY spectrum in 90% H_2_O / 10% D_2_O at 293 K. In order to reduce the size of conformational search space and to achieve a better convergence, the following additional NMR derived restraints were introduced. The results of NOE patterns were used to deduce the hydrogen bond restraints of the secondary structures (i.e. two β-strands and the C-terminal α-helix), which were assigned to 2.8 ± 0.5 Å for r_N-O_ and 1.8 ± 0.5 Å for r_H-O_.

The ^3^*J*_HNHα_ constants were determined from COSY spectra [31] and φ dihedral angles were restrained to the ranges of -120° ± 30° for ^3^*J*_HNHα_ > 8 Hz and -60° ± 30° for ^3^*J*_HNHα_ < 6 Hz.

The ^1^H^α^ and ^13^C^α^ chemical shifts of assigned residues were used to predict the secondary structure restraints. This was done by using the chemical shift indexing method (CSI) [32] along with the information derived from the TALOS program [33].

### Structure calculations

The structural calculations were carried out on an intel based workstation using CNS [34] and the standard protocols of ARIA 1.2 program [35] under CentOS Linux 6.5. A simulated annealing protocol in torsion angle space was used starting from an extended conformation. The protocol was implemented in four stages: i) high temperature simulated annealing at 10000 K; ii) a first cooling phase from 10000 K to 1000 K in 5000 steps; iii) a second cooling phase from 1000 K to 50 K in 4000 steps; and iv) 4000 steps refinement and energy minimization. The time step for integration was set to 0.003 ps. One hundred structures were generated in each iteration, and the 20 lowest energy structures in the final iteration were evaluated using PROCHECK-NMR [36]. The structural models were visualized with program UCSF Chimera [37].

### Molecular modeling of the complex between PPTI and trypsin

The binding structure of PPTI and trypsin was homology modeled according to the crystal structure of BPTI in complex with trypsin by using Modeller [38]. The best complex model was selected based on DOPE score among 1000 generated models.

### Molecular modeling of the 3D structure of human Kv1.1 potassium channel

According to the sequence homology of human Kv1.1 based on BLASTp [39] search against PDB, two types of templates were selected for homology modeling of human Kv1.1 potassium channel; crystal structures of eukaryotic Kv1.2 and bacterial KCSA potassium channels. Again, 1000 constructed models were ranked based on DOPE score.

### Brownian dynamics simulation of the interaction between PPTI and human Kv1.1

The ability of PPTI in recognizing and blocking of human Kv1.1 potassium channel was studied by MacroDox simulation package [40]. We used this package for assigning the titratable groups, solving the linearized Possion-Boltzmann (PB) equation and running BD simulation. In order to compensate the rigidity of PPTI, we did a molecular dynamics (MD) simulation to sample enough solution conformations. The MD simulations were carried out with the GROMACS simulation package version 4.6.5 using the Amber99SB-ILDN force field parameters as implemented in GROMACS [41]. After energy minimization, two succeeding position-restrained MD simulations were performed. First, to set the atomic velocities and adjust the system temperature, an MD simulation was performed for 100 ps at desired temperature and in a constant volume condition (NVT). Second, to adjust pressure and densities, a MD simulation with 1 ns duration was carried out at constant temperature and pressure (NPT). Then, a 250 ns NPT simulation was performed to produce an ensemble of PPTI conformations uniformly sampled every 2.5 ns. The coordinates of PPTI structures from the NPT MD trajectory along with Kv1.1 channel structure from homology modeling were used as the initial structures in BD simulations. The atomic charges of titratable functional groups of amino acid side chains were assigned according to Tanford-Kirkwood method as implemented in MacroDox according to the solvent ionic strength of 0.1 M, temperature of 298 K and pH value of 7.0. The PB equation was solved for two cubic lattices, and the center of mass of Kv1.1 channel as receptor was placed at the center of lattice. The resolutions were set to 0.5 and 0.75 Å for inner and outer grids respectively. At the beginning of each trajectory, the center of mass of PPTI was randomly placed on surface of a sphere with radius of 82 Å that its center is located in the center of simulation lattice. Only PPTI was allowed to rotate and translate and Kv1.1 channel was immobilized in the center of simulation lattice. Whenever the distance between the center of mass of PPTI and Kv1.1 channel exceeded an escape radius of 200 Å, the trial trajectory was terminated. In order to reach a suitable complex model between PPTI and Kv1.1 potassium channel we did 100,000 trial trajectories in each BD simulation.

## Supporting information

S1 Fig NOESY fingerprint. The NOESY fingerprint region of PPTI with the mixing time of 150 ms.
(TIF)

S2 Fig Defined hydrogen bonds of the β-sheet motif. Hydrogen bond pattern observed by long range NOEs.
(TIF)

S3 Fig The modeled structure of human Kv1.1 potassium channel. The side view of human Kv1.1 potassium channel. The water molecule at the pore entry is shown as a green sphere.
(TIF)

S4 Fig The modeled complex of PPTI and Kv1.1 potassium channel. The side view of complexes between PPTI and human Kv1.1 potassium channel. The small blue spheres show the center of mass of PPTI in each successful trajectory of BD simulation. The radii of inner sphere of these BD simulations are 15 Å (A) and 7 Å (B).
(TIF)

S5 Fig The modeled complex of α-DTX and Kv1.1 potassium channel. The side view of complex between α-DTX and human Kv1.1 potassium channel. The orientation of the critical residue, K5, is depicted and labeled. The water molecule at the pore entry is shown as a green sphere.
(TIF)

S6 Fig The electrostatic isocontour potential map and the surface potential of α-DTX. The blue and red colors represent positive and negative fields respectively. The electric dipole moment vector of the protein is represented as a green arrow. The K5 critical residue is labeled.
(TIF)

S1 Table The chemical shift table of PPTI. (PDF)

S2 Table Structural statistics of the twenty best NMR structures. (PDF)
