# Supplementary figures and images for: Structural characterization of PPTI, a kunitz-type protein from the venom of Pseudocerastes persicus
Source: PLoS One. 2019 Apr 11;14(4):e0214657. doi: 10.1371/journal.pone.0214657 (PMC6459475; doi:10.1371/journal.pone.0214657)

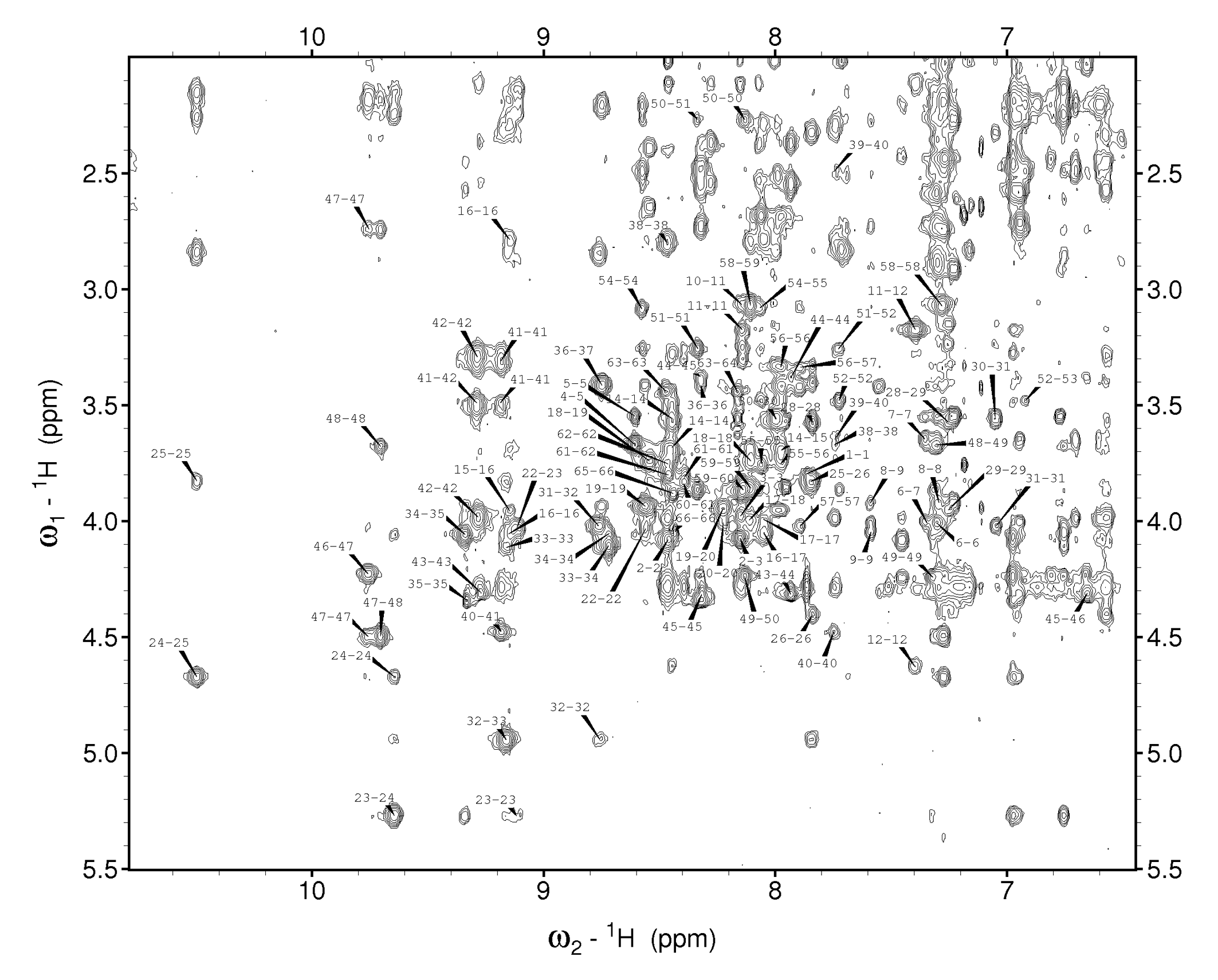

Supplement: S1 Fig — The NOESY fingerprint region of PPTI with the mixing time of 150 ms. (TIF) [file pone.0214657.s001.tif]

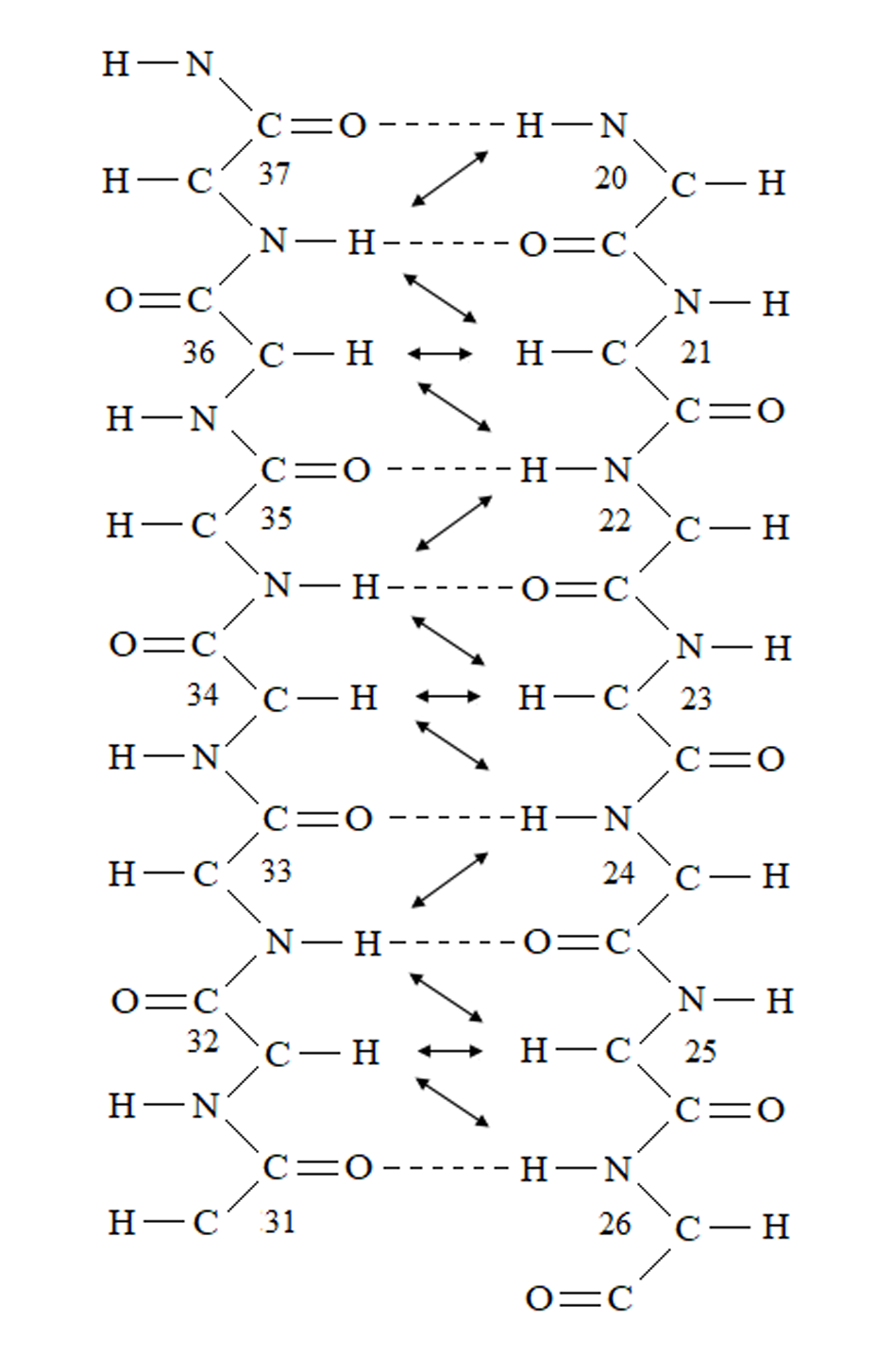

Supplement: S2 Fig — Hydrogen bond pattern observed by long range NOEs. (TIF) [file pone.0214657.s002.tif]

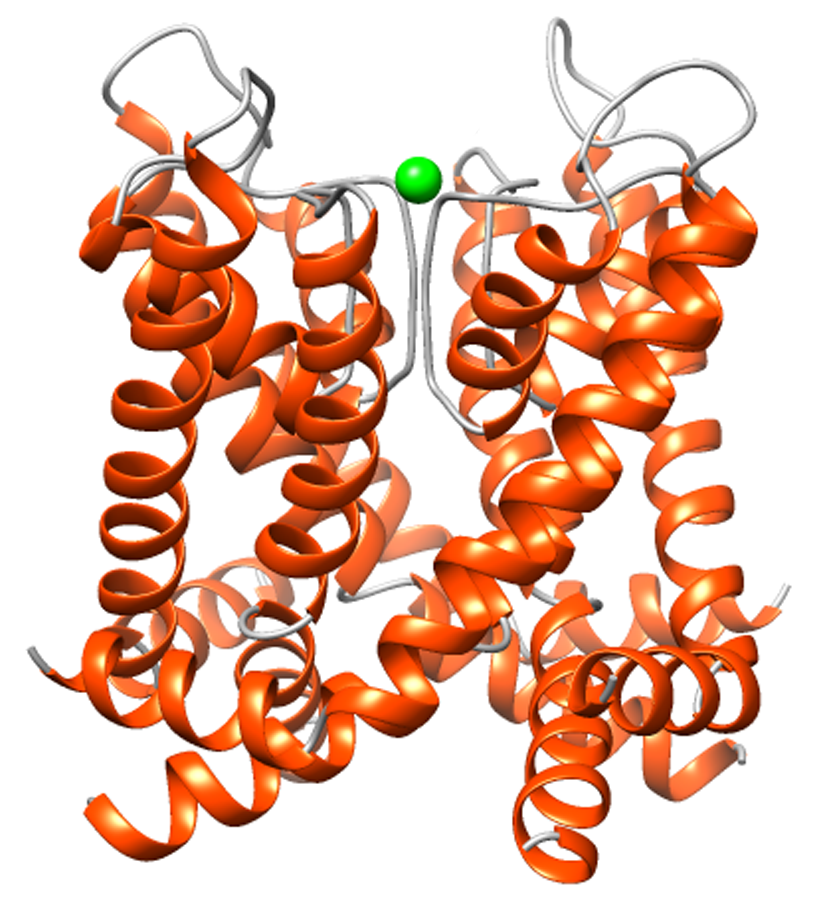

Supplement: S3 Fig — The side view of human Kv1.1 potassium channel. The water molecule at the pore entry is shown as a green sphere. (TIF) [file pone.0214657.s003.tif]

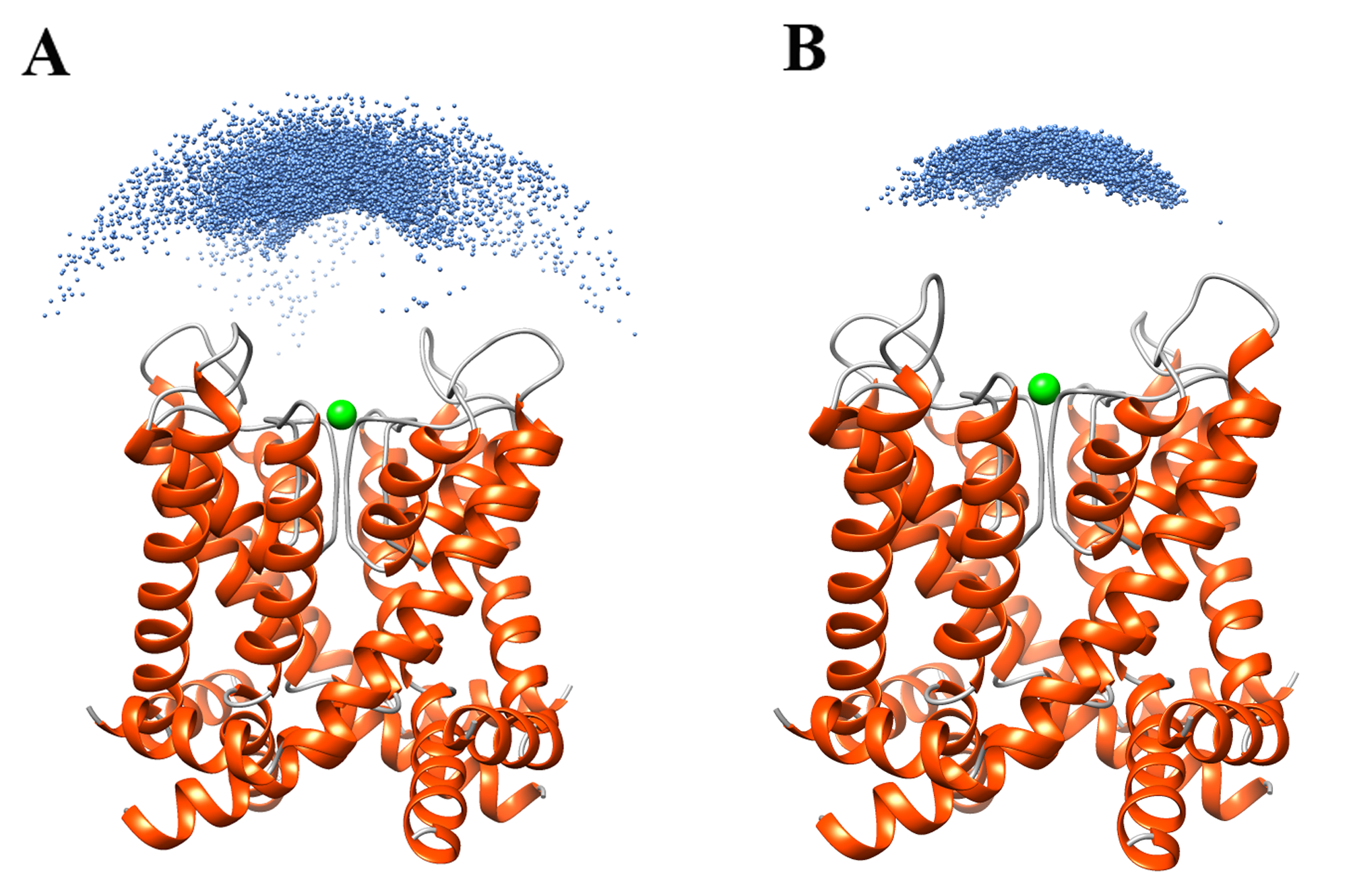

Supplement: S4 Fig — The side view of complexes between PPTI and human Kv1.1 potassium channel. The small blue spheres show the center of mass of PPTI in each successful trajectory of BD simulation. The radii of inner sphere of these BD simulations are 15 Å (A) and 7 Å (B). (TIF) [file pone.0214657.s004.tif]

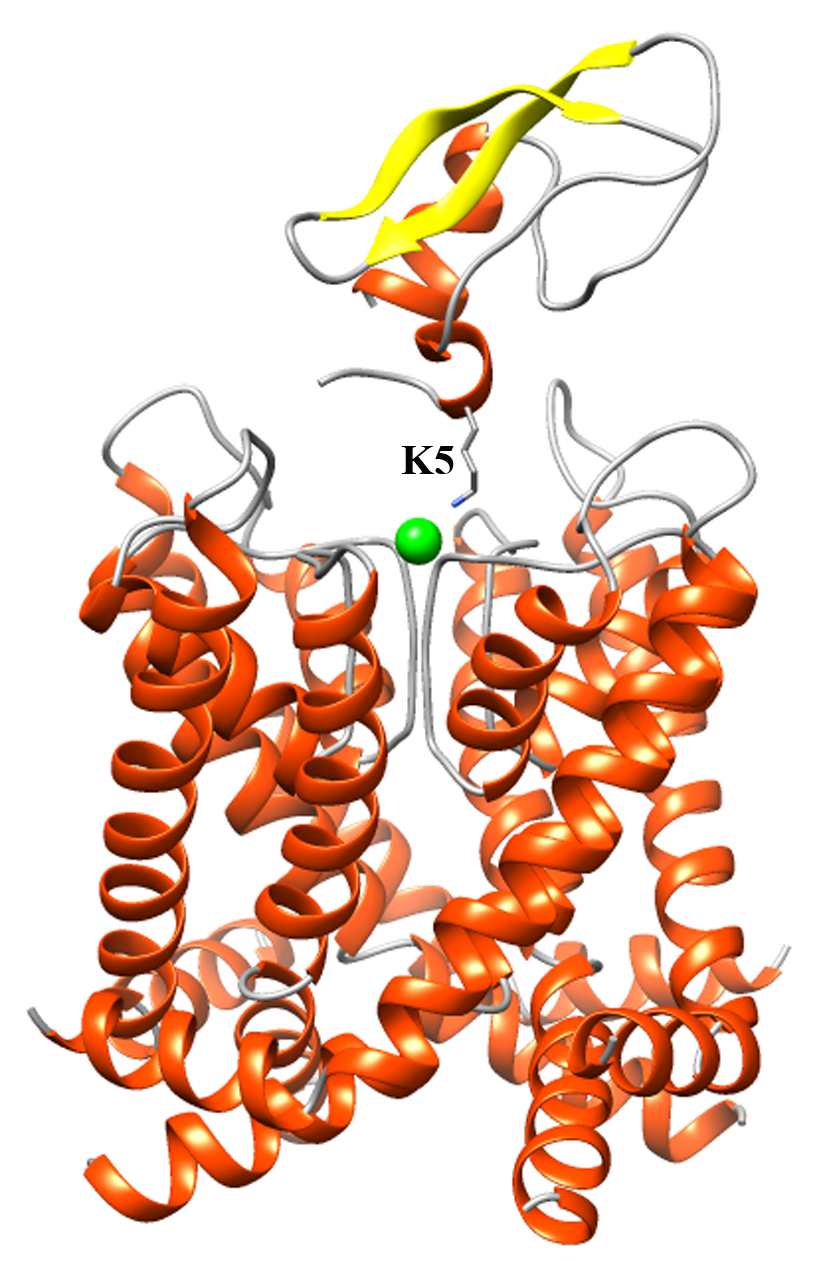

Supplement: S5 Fig — The side view of complex between α-DTX and human Kv1.1 potassium channel. The orientation of the critical residue, K5, is depicted and labeled. The water molecule at the pore entry is shown as a green sphere. (TIF) [file pone.0214657.s005.tif]

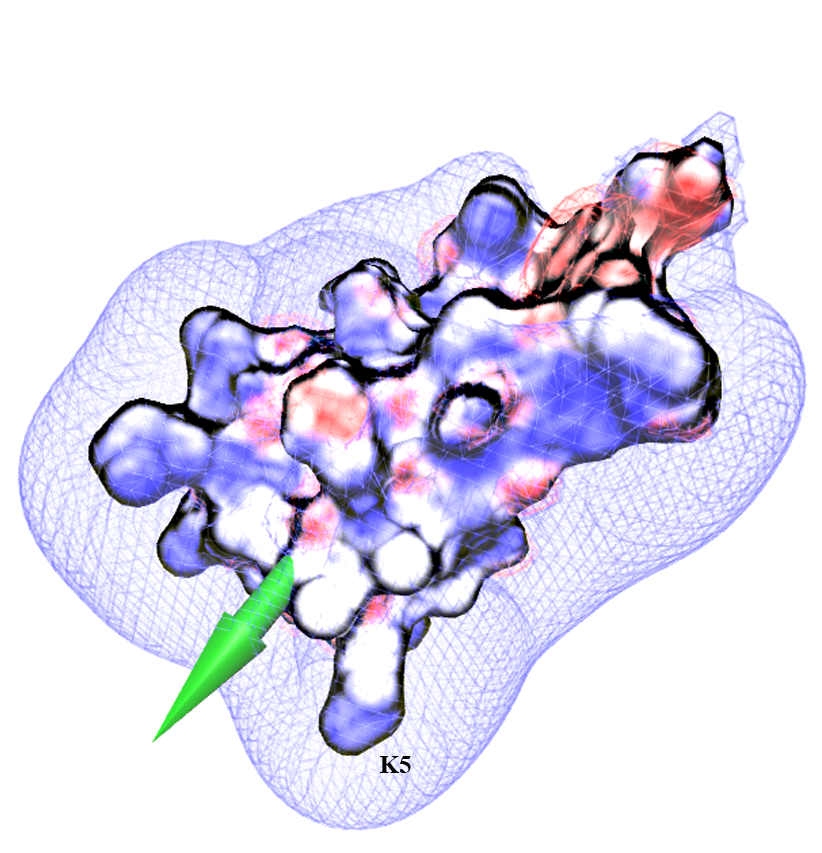

Supplement: S6 Fig — The blue and red colors represent positive and negative fields respectively. The electric dipole moment vector of the protein is represented as a green arrow. The K5 critical residue is labeled. (TIF) [file pone.0214657.s006.tif]
