# Supplementary material for: Structural characterization of PPTI, a kunitz-type protein from the venom of Pseudocerastes persicus
Source: PLoS One. 2019 Apr 11;14(4):e0214657. doi: 10.1371/journal.pone.0214657 (PMC6459475; doi:10.1371/journal.pone.0214657)
Supplement: S1 Table — (PDF) [file pone.0214657.s007.pdf]

S1 Table. The chemical shift table of PPTI.

Pga stands for Pyroglutamic acid. The unassigned chemical shifts are designated by U.

Residue	Chemical Shift (ppm)				
	H^N	H^{α}	H^{β}	Other Protons	C^{α}
**Pga1**	7.865	4.297	1.997	H^{γ} 2.458, 2.335	U
**Asp2**	8.463	4.577	2.610,2.502		55.096
**Arg3**	8.152	4.476	1.602	H^{γ} 1.494	53.853
**Pro4**		4.170	1.901,1.810	H^{γ} 1.601, 0.803; H^{δ} 3.717, 3.542	62.911
**Lys5**	8.607	4.050	1.897	H^{γ} 1.586 ; H^{δ} 1.675	59.173
**Phe6**	7.302	4.508	3.210,3.086	H^{δ} 6.945; H^{ε} 7.335; H^{Z} 7.336	62.397
**Cys7**	7.353	4.147	2.457,2.275		58.150
**Tyr8**	7.296	4.425	3.398,2.765	H^{δ} 7.228; H^{ε} 6.767	58.002
**Leu9**	7.587	4.545	1.972,1.931	H^{γ} 1.572; H^{δ} 1.089, 0.993	52.264
**Pro10**		3.571	1.776	H^{γ} 2.239; H^{δ} 3.806, 3.579	63.428
**Asp11**	8.146	3.673	0.332,0.097		52.930
**Asp12**	7.396	5.125	2.614,2.267		49.604
**Pro13**		4.490	2.895	H^{γ} 2.233, 2.182; H^{δ} 3.989, 3.774	63.006
**Gly14**	8.447	4.188,4.059			44.892
**Val15**	7.975	4.455	2.389	H^{γ} 0.906, 0.830	63.560
**Cys16**	9.147	4.553	3.284,2.792		53.931
**Lys17**	8.050	4.494	2.075	H^{γ} 1.346, 1.213; H^{δ} 1.498; H^{ε} 2.913	58.328
**Ala18**	8.109	4.240	1.151		51.827
**His19**	8.549	4.440	3.138,2.897	$H^{\delta 2}$ 7.112; $H^{\varepsilon 1}$ 8.488	57.781
**Ile20**	8.226	4.508	1.871	H^{γ} 1.310, 0.884; H^{δ} 0.629	60.425
**Pro21**		4.434	2.184	H^{γ} 1.775; H^{δ} 3.916	62.247
**Arg22**	8.565	4.530	1.634	H^{γ} 0.624; H^{δ} 7.303, 6.509	54.331
**Phe23**	9.114	5.771	2.746,2.639	H^{δ} 6.755; H^{ε} 7.323; H^{ζ} 7.321	57.200
**Tyr24**	9.644	5.173	2.751,2.667	H^{δ} 6.952; H^{ε} 6.576	55.215
**Tyr25**	10.497	4.329	3.337,2.651		59.416
**Asn26**	7.842	4.904	2.825,1.941		49.451
**Pro27**		3.827	2.173	H^{γ} 2.304; H^{δ} 4.048, 3.904	64.608
**Ala28**	7.840	4.075	1.400		54.888
**Ser29**	7.254	4.450	3.736,3.649		61.851
**Ans30**	7.999	4.059	3.331,2.508	H^{δ} 7.715, 7.164	53.789
**Lys31**	7.050	4.518	1.615	H^{γ} 1.273; H^{δ} 1.411	55.828
**Cys32**	8.762	5.443	3.351,2.440		57.999
**Lys33**	9.162	4.613	1.810	H^{γ} 1.203; H^{δ} 1.554	54.654
**Glu34**	8.719	4.554	1.822	H^{γ} 1.946	53.450
**Phe35**	9.338	4.842	3.076,2.955	H^{δ} 6.973; H^{ε} 6.972; H^{ζ} 6.809	55.381
**Ile36**	8.321	3.913	1.867	$H^{\gamma 1}$ 1.260, 1.154; $H^{\gamma 2}$ 0.371; H^{δ} 0.334	58.151
**Tyr37**	8.749	4.581	2.706,2.231	H^{δ} 7.454, 6.602; H^{ε} 6.703, 6.564	55.803
**Gly38**	8.466	4.178,3.295			45.600
**Gly39**	U	4.140,2.983			45.432
**Cys40**	7.744	4.977	2.804,3.100		54.112
**Gly41**	9.184	3.986,3.815			45.796
**Gly42**	9.287	4.482,3.788			43.770
**Asn43**	9.274	4.811	3.036,2.870	H^{δ} 8.279, 8.072	51.611
**Ala44**	7.934	3.883	0.576		52.871
**Asn45**	8.321	4.814	3.221,3.006	H^{δ} 7.991, 7.692	50.685
**Asn46**	6.657	4.734	2.527,2.414		53.439
**Phe47**	9.757	4.996	3.240,2.688	H^{δ} 7.275; H^{ε} 7.273; H^{ζ} 7.553	55.863
**Glu48**	9.704	4.174	2.239	H^{γ} 2.448	59.437
**Thr49**	7.312	4.741	4.366	H^{γ} 1.221	U
**Arg50**	8.131	2.769	1.216,1.058	H^{γ} 0.906, 0.703; H^{δ} 3.092, 2.988	59.469
**Ala51**	8.335	3.755	1.252		55.179
**Glu52**	7.725	3.979	2.085	H^{γ} 2.370	58.749
**Cys53**	6.919	1.799	2.988,2.716		59.002
**Arg54**	8.572	3.582	1.713,1.634	H^{γ} 1.365; H^{δ} 3.051	59.171
**His55**	8.063	4.254	3.183	$H^{\delta 2}$ 8.384; $H^{\varepsilon 1}$ 7.183	58.051
**Thr56**	7.972	3.835	3.918	H^{γ} 1.502	66.094
**Cys57**	7.894	4.522	2.093,1.810		57.164
**Val58**	7.284	3.572	2.166	H^{γ} 0.856, 0.786	60.159
**Ala59**	8.109	4.361	1.245		51.842
**Ser60**	8.134	4.337	3.806,3.750		58.608
**Arg61**	8.393	4.305	1.783,1.676	H^{γ} 1.551; H^{δ} 3.107; H^{ε} 7.213	55.926
**Lys62**	8.456	4.253	1.788	H^{γ} 1.392; H^{δ} 1.689; H^{ε} 2.929	56.509
**Gly63**	8.472	3.942			44.938
**Gly64**	8.164	4.082,4.029			44.366
**Pro65**		4.381	2.243,1.839	H^{γ} 1.941; H^{δ} 3.554	63.402
**Arg66**	8.434	4.543	1.778	H^{γ} 1.672; H^{δ} 3.154; H^{ε} 7.187	54.070
**Arg67**	7.936	U	U	H^{ε} 7.156	57.565
**Pro68**		U	U		U
