# Supplementary material for: Structural characterization of PPTI, a kunitz-type protein from the venom of Pseudocerastes persicus
Source: PLoS One. 2019 Apr 11;14(4):e0214657. doi: 10.1371/journal.pone.0214657 (PMC6459475; doi:10.1371/journal.pone.0214657)
Supplement: S2 Table — (PDF) [file pone.0214657.s008.pdf]

S2 Table. Structural statistics of the twenty best NMR structures.

Parameter	Value
Distance restraints	
Intra residue *(i-j = 0)*	279
Sequential *(|i-j| = 1)*	195
Medium range *(|i-j| < 5)*	72
Long range *(|i-j| > 5)*	212
Hydrogen bonds	10
Total	768
Dihedral-angle restraints	
$^{3}J_{NH\alpha}$	14
Secondary chemical shifts	27
Mean RMS deviation from experimental restraints	
NOE (A°)	0.18 ± 0.002
Dihedrals (°)	0.56 ± 0.10
Mean RMS deviation from idealized covalent geometry	
Bonds (A°)	0.00 ± 0.00
Angles (°)	0.50 ± 0.06
Improper (°)	0.41 ± 0.11
Mean energy (kcal.mol^{-1})	
E_{NOE}	49.51 ± 14.41
E_{dih}	338.05 ± 8.38
E_{vdw}	-578.70 ± 8.45
E_{bond}	12.98 ± 4.90
$E_{improper}$	16.64 ± 8.88
E_{angle}	76.65 ± 19.28
E_{elec}	-1825.71 ± 69.71
E_{total}	-1960.08 ± 97.65
PROCHECK Ramachandran plot analysis for the best 20 structures	
Most favored region (%)	69.5
Additionally allowed region (%)	29.2
Generously allowed region (%)	0.8
Disallowed region (%)	0.5

	Atomic RMS differences (A°)	
Residues	Backbone atoms	Heavy atoms
1-68	4.0	4.9
1-58 (excluding C-terminal floppy tail)	0.9	1.4
